# Bromide of Potassium as a Caustic

**Published:** 1877-06

**Authors:** 


Bromide of Potassium as a Caustic.—In a paper read at the recent meeting of the French Association for the Advancement of Science, M. Peyrand, of Dibourne, claims for bromide of potassium certain properties hitherto but slightly recognized—properties which will extend the already wide range of the therapeutical uses of this salt. He found that subcutaneous injection in rabbits of concentrated solutions of the salt led to sloughing of the skin, and from this he was led to try the value of what he considered to be the escharotic properties of bromide of potassium upon malignant and other growths, either by means of injections into the tumour or by the application of the powdered salt to a raw surface. The action of the salt is completely resisted by the tegument. His first clinical experiment on the subject took place in April, 1874, when, by means of daily applications of powdered bromide, he affected the removal within twenty-eight days of an epitheliomatous growth on the face. He has since had equally good results from this treatment of atonic ulcers of the legs, rapid cicatrization following the separation of sloughs produced by the application. In such eases he uses either the powder or an ointment of one part in five, or a mixture (one in ten) of glycerine and the bromide. In many skin affections, as chronic eczema, pityriasis, and acne, in phagedæna, ulcerative stomatitis, and many other local inflammatory disorders, he has found it of use. As a local hæmostatic, a solution of one in fifty has served for epistaxis, and as a general hæmostatic its success in many cases of hæmoptysis and metrorrhagia was very marked, where ergot, perchloride of iron, and rhatany had failed. —*Lancet*, Sept. 30, 1876.

